# Text clustering based on pre-trained models and autoencoders

**DOI:** 10.3389/fncom.2023.1334436

**Published:** 2024-01-05

**Authors:** Qiang Xu, Hao Gu, ShengWei Ji

**Affiliations:** School of Artificial Intelligence and Big Data, Hefei University, Hefei, Anhui, China

**Keywords:** text clustering, medical, deep learning, pre-trained models, autoencoder, deep embedded clustering model

## Abstract

Text clustering is the task of grouping text data based on similarity, and it holds particular importance in the medical field. sIn healthcare, medical data clustering is a highly active and effective research area. It not only provides strong support for making correct medical decisions from medical datasets but also aids in patient record management and medical information retrieval. With the development of the healthcare industry, a large amount of medical data is being generated, and traditional medical data clustering faces significant challenges. Many existing text clustering algorithms are primarily based on the bag-of-words model, which has issues such as high dimensionality, sparsity, and the neglect of word positions and context. Pre-trained models are a deep learning-based approach that treats text as a sequence to accurately capture word positions and context information. Moreover, compared to traditional K-means and fuzzy C-means clustering models, deep learning-based clustering algorithms are better at handling high-dimensional, complex, and nonlinear data. In particular, clustering algorithms based on autoencoders can learn data representations and clustering information, effectively reducing noise interference and errors during the clustering process. This paper combines pre-trained language models with deep embedding clustering models. Experimental results demonstrate that our model performs exceptionally well on four public datasets, outperforming most existing text clustering algorithms, and can be applied to medical data clustering.

## 1 Introduction

With the rapid development of big data in the healthcare industry, there has been a dramatic increase in the amount of available medical text information generated, which covers many forms of data such as medical records, clinical reports, scientific literature, medical forum posts and patient feedback, and much of it is unstructured, which is a serious challenge in how to use this information (Woo et al., 2019). Recently, with considerable advances in new neural networks and deep learning methods for natural language processing (NLP) (Li et al., 2022), the application of natural language processing in the field of medicine has received increasing attention, and text clustering is one of the important techniques to help process the huge amount of unstructured text data in the medical field that contains valuable medical information. By grouping similar text documents into the same category, text clustering technology helps healthcare practitioners and researchers to better understand, manage, and utilize this information. Bu et al. (2020) has developed a C-means algorithm based on a cloud-edge computing system, which enables the aggregation of medical data from different hospitals. Jaya Mabel Rani and Pravin (2022) has proposed a hybrid optimization technique based on K-means, effectively assisting doctors in aggregating medical data related to heart disease to find the best solution.

According to prior research, text clustering essentially involves categorizing texts based on their similarity in effective representations, comprising two primary modules: text feature representation and clustering (Aggarwal and Zhai, 2012). A common approach in text clustering is to map texts into a feature vector space and then employ clustering algorithms. Although traditional TF-IDF (Ramos et al., 2003) methods are capable of representing real-valued vectors, they fail to capture textual sequence and contextual information, particularly for shorter texts where sparse features hinder semantic inference. Word2Vec (Mikolov et al., 2013), a word embedding-based technique, employs neural networks to learn continuous vector representations for each word, thus capturing semantic relationships and contextual information between vocabulary words, effectively addressing issues posed by the bag-of-words model. Nonetheless, these models still rely on feature space and do not resolve the problem of semantic understanding discrepancies. In recent years, text representation models rooted in deep learning, such as BiLSTM (Conneau et al., 2017) and BERT (Devlin et al., 2018), have gained widespread application. They excel in handling text sequences and contextual information, making them well-suited for text clustering tasks.

A clustering algorithm is employed to partition a document collection into distinct clusters or categories. Documents within the same cluster should exhibit maximum similarity, while those in different clusters should be as dissimilar as possible (Bhattacharjee and Mitra, 2021). Traditional clustering methods encompass K-means, hierarchical clustering, and density-based clustering. Over the past years, an increasing number of researchers have delved into the integration of deep learning-based text representation models with conventional clustering algorithms. Xu et al. (2017) proposed a deep short text clustering method using RecNN+K-means. However, this model pretrains a convolutional neural network on unlabeled short text data to learn word co-occurrences, which might lead to word representations not aligning well with their actual semantics. Guan et al. (2020) introduced a BiLSTM+K-means deep clustering framework, which exhibited improvement over BERT+K-means. Yin et al. (2023) proposed a method that combines SentenceBERT model with improved k-means algorithm, which improves the efficiency of clustering scientific and technological literature bibliographic information, so that we can better obtain the key information of scientific and technological literature. However, K-means is sensitive to outliers and can be disrupted by them. Furthermore, as data dimensionality increases, calculating distances between samples becomes progressively challenging, resulting in diminished clustering effectiveness. Deep clustering algorithms based on autoencoders obviate the need for preset cluster centers, autonomously learn data representations, and perform clustering, thereby mitigating reliance on initial values. Moreover, these algorithms can use autoencoders to perform dimensionality reduction, addressing not only high-dimensional clustering but also removing noise and outlier interference (Xie et al., 2016). High-dimensional sparsity refers to the fact that when dealing with text data, due to the large number of features and wide distribution of samples, most features do not contribute to the model and only produce noise signals. This characteristic makes the data appear sparse in high-dimensional space, and the similarity between any two samples is close to zero. Noise can be considered as random errors or changes added to the original signal. When dealing with text data, various types of noise may be encountered, such as spelling errors, grammar errors, stop words, etc.

**Figure 1:**
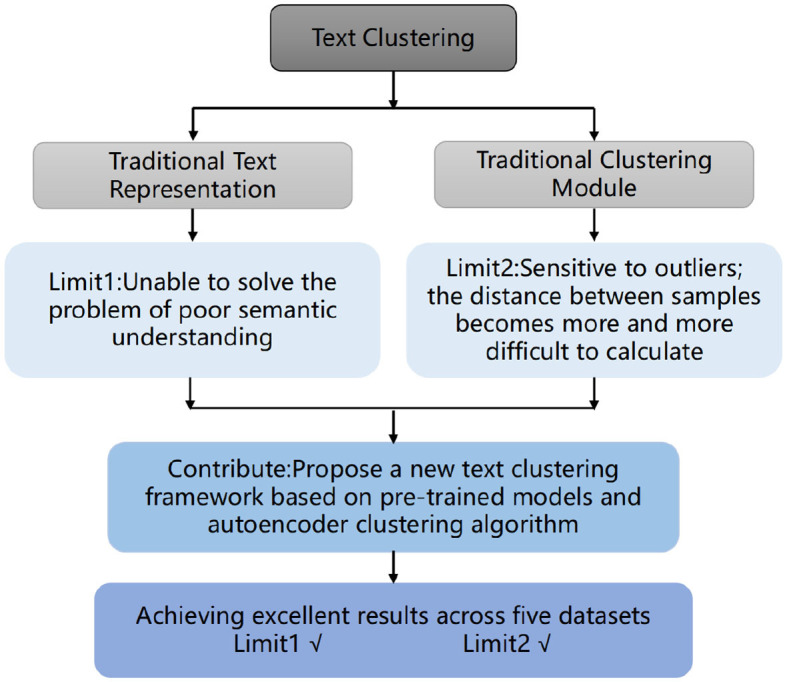
Relationship between motivation and contribution in this paper.

Hence, we propose a novel deep learning-based text clustering framework named pre-trained Encoder-Encoder Clustering (TCBPMA), by amalgamating deep pre-trained language models with autoencoder-based deep clustering algorithms. As shown in the Figure 1, this framework enhances text clustering from two facets: text vectorization and clustering. In contrast to feature-based text representation methods, pre-trained deep encoders offer enriched semantic representations and alleviate feature sparsity issues. Building on this foundation, we leverage the autoencoder in the deep clustering algorithm for feature learning and clustering, further elevating clustering effectiveness. Moreover, as both models rely on deep learning techniques, they inherently possess scalability. When confronted with large-scale text data, improvements in clustering can be achieved by augmenting training data and adjusting model parameters. We benchmark our model against classic text clustering models, and experimental results demonstrate its superiority across nearly all considered datasets.

Our contributions in this study are as follows:

1. We introduce autoencoder-based clustering algorithms to short text clustering, proposing a novel deep learning-based text clustering framework (TCBPMA). By combining pre-trained models and autoencoder clustering algorithms, we capture text semantic features more accurately, leading to improved clustering results.
2. Extensive experiments (on five datasets, with seven comparative methods, and using three different metrics) demonstrate that the proposed framework significantly outperforms the SOTA competitor.
3. We organize a novel dataset containing content from government complaint phone calls, applying our framework and achieving favorable results. Furthermore,we can better understand and address real-world government complaints, improve problem-solving efficiency, and provide the government with more informed recommendations for policies and services.

## 2 Related work

We categorize related work into three main domains: the first introduces existing deep feature extraction methods, the second discusses the BiLSTM-based ELMO model, and the third covers autoencoders.

### 2.1 Deep learning-based feature extraction methods

Deep learning-based feature extraction methods involve employing deep neural networks to learn representations of input data in order to better capture data features and structures. Deep learning models use multiple layers of nonlinear transformations to gradually abstract and refine useful information within the data, generating higher-level representations (Dara and Tumma, 2018). Neurons are the fundamental units of deep neural networks, receiving input signals and generating output signals by learning neuron strategies. Analogous to the structure of the brain, raw information is progressively processed and feature-extracted through interconnected hidden neuron layers. Deep neural networks utilize numerous neurons to process input data, attaining higher-level representations and abstractions.

Convolutional Neural Networks (CNNs) (Kowsari et al., 2019) are widely used deep learning models in NLP. Comprising convolutional layers, pooling layers, and fully connected layers, CNNs efficiently extract local features from images. Pooling layers reduce feature dimensions while maintaining positional invariance, and fully connected layers map the extracted features to the final output. Recurrent Neural Networks (RNNs) (Kowsari et al., 2019) are deep learning models suited for sequential data. Introducing recurrent connections within the network enables RNNs to handle temporal information within sequences. At each time step, an RNN receives input and the hidden state from the previous time step, allowing it to capture contextual information and extract relevant features. Jia et al. (2022) proposed a feature extraction method based on Doc2Vec and CNN. The sentence vector was obtained through the DM model training of Doc2Vec and used as the input of CNN to finally obtain the text feature vector. Long Short-Term Memory (LSTM) (Yu et al., 2019) networks are a variation of RNNs that address issues of gradient vanishing and exploding by introducing mechanisms such as memory cells, input gates, forget gates, and output gates. Wan et al. (2020) proposed a short text clustering model ST-CNN. BiLSTM+CNN was used to mine the context information of short texts and obtain deep semantic text feature vectors.

### 2.2 ELMO model

**Figure 2:**
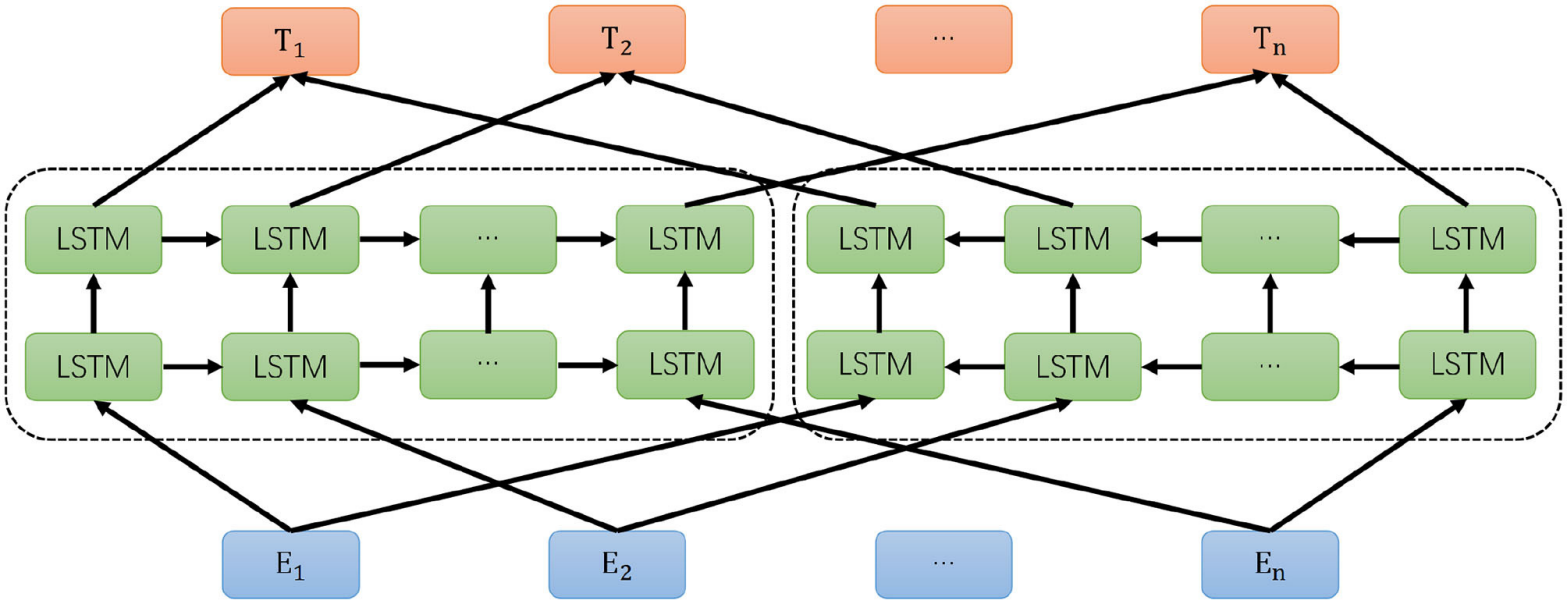
Architecture diagram of ELMO model.

The ELMO model is essentially a two-layer bidirectional LSTM language model (Peters et al., 2018). The forward language model learns the semantic information of a word within its following context, and similarly, the backward language model learns the semantic information of the word within its preceding context. The objective function of the entire model involves maximizing the likelihood of both forward and backward language models. The structure is illustrated in Figure 2.

ELMO consists of three main modules: the bottom blue module is the Embedding module, the middle green module consists of a dual-layer LSTM, and the top orange module is the word vector representation module. The Embedding module employs CNN to encode the character-level model, essentially obtaining a word embedding vector as the network's bottom-layer input. The dual-layer LSTM module is divided into a forward LSTM network and a backward LSTM network. For the forward LSTM, given N tokens (*t*_1_, *t*_2_, …, *t*_*n*_), the Language Model calculates the probability of the k-th token appearing based on the token sequence of the previous k-1 positions, forming the forward dual-layer LSTM model, and the same principle applies to the backward LSTM. The formula for the forward LSTM model is:


(1) $$\begin{array}{l} p\left(t_{1},t_{2},...,t_{n}\right)=\overset{n}{\underset{k=1}{\prod }}p\left(t_{k}|t_{1},t_{2},...,t_{k-1}\right) \end{array}$$

The formula for the backward LSTM model is:


(2) $$\begin{array}{l} p\left(t_{1},t_{2},...,t_{n}\right)=\overset{n}{\underset{k=1}{\prod }}p\left(t_{k}|t_{k+1},t_{k+2},...,t_{n}\right) \end{array}$$

The objective function is to maximize the log likelihood of both forward and backward probabilities, expressed as follows:


(3) $$\begin{array}{l} \overset{n}{\underset{k=1}{\sum }}\left(\log p\left(t_{k}|t_{1},t_{2},...,t_{k-1}\right)+\log p\left(t_{k}|t_{k+1},t_{k+2},...,t_{n}\right)\right) \end{array}$$

By using the above formula, we can obtain a token's forward vector and backward vector, which are then combined to form the token's final output vector (Merity et al., 2017). The ELMO language model is designed to dynamically update word representation vectors to address the issue of polysemy in different contexts. The core idea of ELMO is as follows: Initially, traditional language models are employed to train word embeddings on a large corpus. These static word embeddings cannot distinguish polysemous meanings. Subsequently, the trained data is used with the ELMO model to capture context information, yielding dynamic word embeddings based on the contextual information.

Deep representation learning is conducted using the two-layer bidirectional LSTM language model of ELMO. Dynamic word embeddings obtained through training with the ELMO language model offer several advantages over traditional static word embeddings. First, ELMO's word embeddings are context-specific and dynamic, allowing for different representations of the same word in different contexts. This capability better captures the relationships between words and their contexts. ELMO's word embeddings result from the summation of the outcomes of the bidirectional language model. In supervised NLP tasks, ELMO embeddings can be directly appended to the word vector input of task-specific models or their highest-level representations.

### 2.3 Clustering algorithm based on autoencoder

Autoencoders are a type of unsupervised learning neural network model used for feature extraction and reconstruction of data. They work by compressing input data into a lower-dimensional encoding space and then attempting to reconstruct the input data from this encoding space, with the goal of learning useful features present in the data. An autoencoder consists of two parts: the encoder and the decoder. The encoder maps the input data to a lower-dimensional encoding representation, also known as the latent representation. The decoder maps the encoding representation from the latent space back to the reconstructed data, aiming to restore the original input as closely as possible. The structure is depicted above.

**Figure 3:**
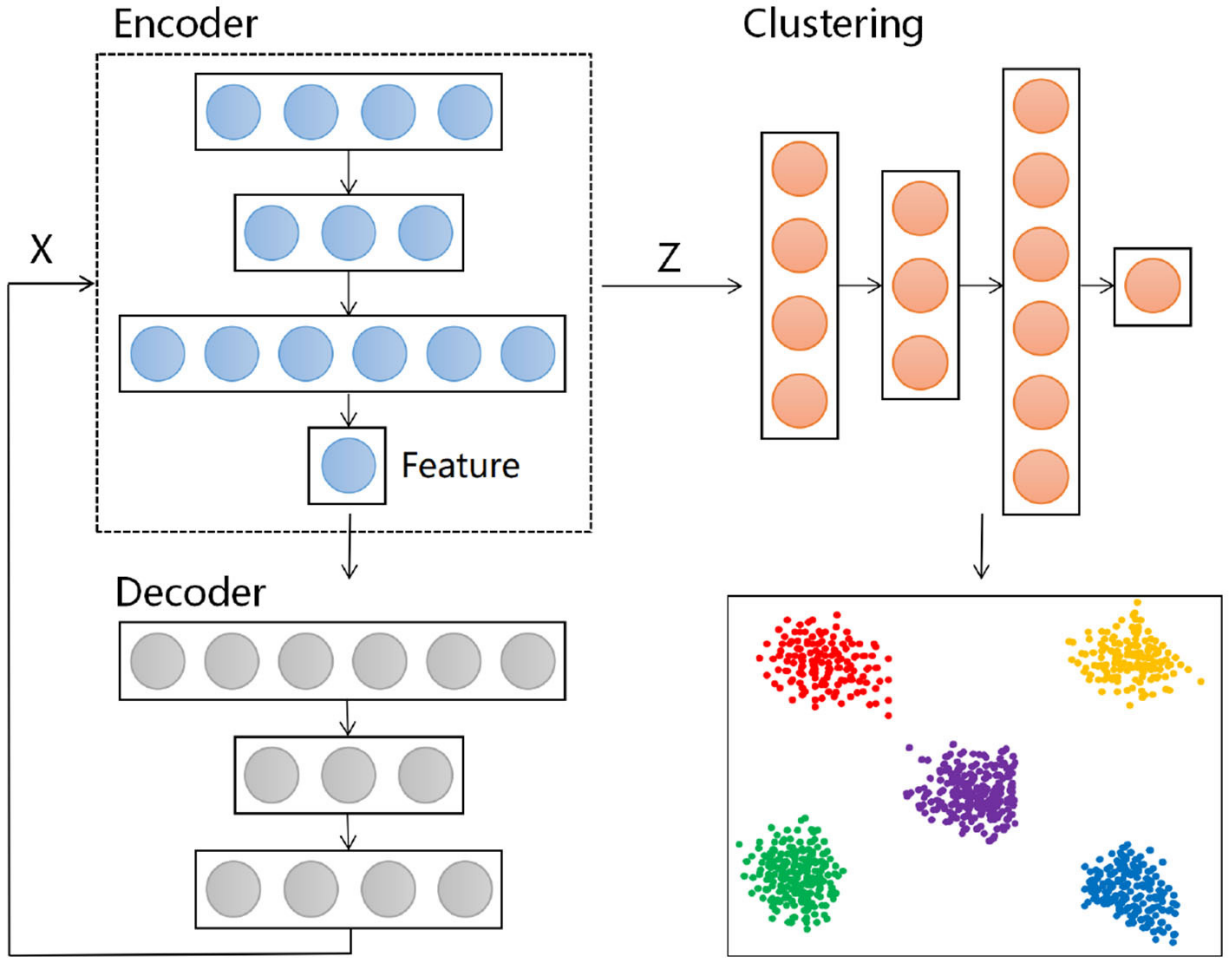
Architecture diagram of DEC model.

DEC (Deep Embedded Clustering) is a clustering algorithm based on autoencoders (Xie et al., 2016). It iteratively learns data vector representations and performs clustering tasks. Assuming we have a dataset with n data points {*x*_1_, *x*_2_, …, *x*_*n*_} and the task is to divide these data points into k clusters, each represented by a center μ_*j*_, where j = 1,…,k. Unlike traditional clustering methods, DEC first applies a nonlinear mapping to the data, transforming it from the feature space X to the latent feature space Z, where θ is the parameter to be learned. This mapping process is parameterized using a neural network structure. The DEC model consists of an encoder and a clustering layer. The DEC algorithm is divided into two main stages: first, the initialization of the autoencoder θ and cluster centers μ_*j*_, forming the foundation of the entire process. Then, it enters the second stage, optimizing θ and μ_*j*_ simultaneously to obtain more accurate data representations and more stable clustering results. This approach, which combines autoencoders and clustering, has significant advantages, particularly suitable for handling high-dimensional data and complex nonlinear data distributions. The model framework is shown in the Figure 3.

## 3 Proposed model

**Figure 4:**
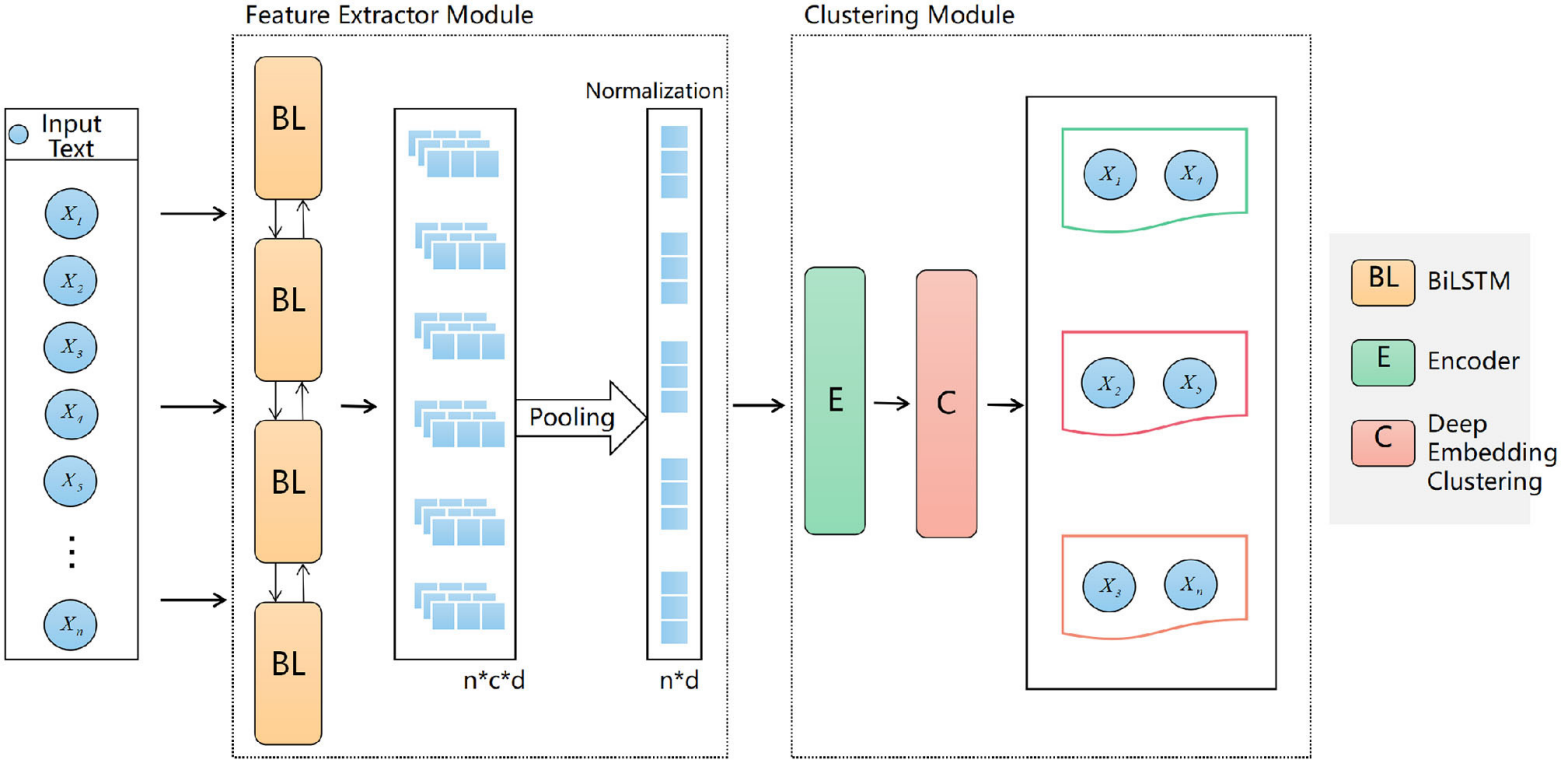
Proposed modeling framework.

Our model framework figure as shown in Figure 4. Given a corpus *D* = {*x*_1_, *x*_2_, …, *x*_*n*_}, where each *x*_1_ is a piece of text, our objective is to partition the texts into several clusters. For each text *x*_1_, we first employ the Bi-LSTM-based ELMO model for feature extraction. Normalization techniques are then applied to ensure feature stability and adherence to a normal distribution. Finally, the normalized feature vectors are fed into the selected clustering algorithm, DEC, to produce results.

### 3.1 Feature extractor module

We train text representation vectors using the ELMO model based on the bidirectional LSTM language model. The language model aims to estimate the probability function of word sequences from a large unlabeled corpus. Given a sequence of n words [*h*_1_, *h*_2_, …, *h*_*n*_] in a text, the pre-trained language model transforms it into vectors [*h*_1_, *h*_2_, …, *h*_*n*_]. Due to the variability in sentence or document lengths, we can't directly input the context features [*h*_1_, *h*_2_, …, *h*_*n*_] into subsequent modules. Thus, it's necessary to fuse the context features and the text representation vectors into fixed-size feature vectors.n this paper, we employ two pooling strategies:

(1) Max Pooling (Hirschberg and Manning, 2015): Max pooling selects the maximum value on each dimension of the d-dimensional context feature vectors [*h*_1_, *h*_2_, …, *h*_*n*_] to construct the text representation. The figure below illustrates how max pooling considers the highest value as the most significant feature.


(4) $$\begin{array}{l} h\left[k\right]=\underset{i=1...n}{max}h_{ik} \end{array}$$

(2) Average Pooling (Huang et al., 2019): The d-dimensional context feature vectors [*h*_1_, *h*_2_, …, *h*_*n*_] are averaged to obtain the feature vector. The concept behind average pooling is that all context feature vectors contribute to representing the entire text, and the average of these vectors reduces noise in the model. The formula is illustrated below.


(5) $$\begin{array}{l} h\left[k\right]=\frac{1}{n}\underset{i=1...n}{\sum }h_{ik} \end{array}$$

After feature extraction, we perform feature normalization on the vector representations obtained from the feature extraction strategies. Feature normalization is essential to ensure that fixed-size vector representations possess normality or stability. We utilize four normalization strategies: identity normalization, standard normalization, layer normalization, and min-max normalization. In this paper, we select layer normalization as the baseline experiment for comparison with other strategies.

(1) Identity Normalization (Guan et al., 2020): Identity normalization is a unit function, defined by the formula:


(6) $$\begin{array}{l} f\left(h\right)=h \end{array}$$

(2) Standard Normalization (Guan et al., 2020): Standard normalization is a common feature normalization method. It transforms input feature vectors into vectors with a unit norm using the following formula. After transformation, the Euclidean distance between two feature vectors equals their cosine distance.


(7) $$\begin{array}{l} h_{i}=\frac{h_{i}}{\left||h_{i}|\right|} \end{array}$$

(3) Layer Normalization (Ba et al., 2016): Layer normalization is a strategy that helps mitigate the problem of covariate shift during neural network training. The normalization layer applies the function shown in the figure below.


(8) $$\begin{array}{l} h_{i}=\frac{h_{i}-\varphi_{i}}{\sigma_{i}} \end{array}$$

(4) Min-Max Normalization (Han et al., 2022): Min-max normalization is a strategy that maintains the original distribution of feature vectors while normalizing. The formula is as follows:


(9) $$\begin{array}{l} h_{i}=\frac{h_{i}-{\text{min}}_{d}\left(h_{id}\right)}{{\text{max}}_{d}\left(h_{id}\right)-{\text{min}}_{d}\left(h_{id}\right)} \end{array}$$

### 3.2 Clustering module

First, we use the feature-extracted vectors as input to train the autoencoder component. The objective of the autoencoder is to minimize the error between the input and its reconstruction, thereby learning a compact representation of the data. In this stage, the autoencoder's weight parameters are updated using backpropagation. After training, we extract the output from the encoder, which represents the latent feature vectors. These vectors serve as the new representation of the data features. Subsequently, we initialize the feature vectors using k-means clustering to create the initial cluster centers. Based on the current cluster centers, we assign the feature vectors to the nearest cluster. Here, we use a student-t distribution to measure the similarity between embedded nodes and cluster centers, as expressed by the following formula:


(10) $$\begin{array}{l} q_{ij}=\frac{{\left(1+\frac{||z_{i}-\mu_{i}|{|}^{2}}{\alpha }\right)}^{\frac{\alpha +1}{2}}}{\underset{j^{{\;}^{\prime }}}{\sum }{\left(1+\frac{||z_{i}-\mu_{i}|{|}^{2}}{\alpha }\right)}^{\frac{\alpha +1}{2}}} \end{array}$$

Where *z*_*i*_ is the embedded vector of *x*_*i*_ after mapping, α represents the degrees of freedom, which we set to 1. *q*_*ij*_ is the probability of assigning sample i to cluster j. Subsequently, we use the minimization of KL divergence as the objective function, as shown in the following formula:


(11) $$\begin{array}{l} L=KL\left(P||Q\right)=\underset{i}{\sum }\underset{j}{\sum }p_{ij}\log\frac{p_{ij}}{q_{ij}} \end{array}$$

Where *p*_*ij*_ is the auxiliary distribution of *q*_*ij*_. Based on the above, we update the cluster centers to minimize the squared error within the clusters. Finally, we iterate through these steps until reaching the convergence criteria or the maximum iteration limit.

Our parameter settings are as follows: The neural network architecture follows the default settings in the original paper (Xie et al., 2016). The maximum number of iterations for pretraining is set to 500. During the optimization phase, the values for α, batch size, and update interval are all the same, which are 1, 256, and 30, respectively. The graphics card we used is NVIDIA-GeForce-RTX-3090, the cuda version is 11.6, the pytorch version is 1.12.0, and the code tool is Pycharm.

## 4 Experiments

### 4.1 Comparison method

**TF-IDF:** TF-IDF is a weighted calculation representing the importance of a word in a text. TF represents the frequency of a word in the text, where a higher TF value implies greater representativeness. For instance, in a text centered around “mobile phones,” the word “mobile” would have a high frequency and strong representation. However, frequently used words like “of,” “you,” and “I” also have high frequencies. Therefore, relying solely on TF values might not accurately gauge a word's importance in the text. To address this, this paper introduces IDF to measure words. When a word is frequently used in the current text but less so in other texts, its IDF value is higher, indicating greater importance to the current text.

**Word2Vec:** Word2Vec is a neural network-based word vector generation model designed to map words to low-dimensional vectors for use in machine learning algorithms. It learns by analyzing extensive text data and employs two models, Skip-gram and CBOW, to generate vector representations for each word. Word2Vec's central idea is to predict a word's probability based on its context (other words in a text), thus learning word vector representations. These vectors have strong semantic and contextual relevance, making them suitable for various natural language processing tasks.

**BERT:** BERT, short for Bidirectional Encoder Representation from Transformers, is a pre-trained model based on Transformers. Researchers designed the BERT model using Transformers as a foundation and trained it on a large corpus through two tasks. The first task involves masking certain words in the text and predicting them based on the context of the other unmasked words. The second task predicts whether two sentences are consecutive. Through these tasks, the BERT pre-trained model excels in capturing both semantic and contextual information from text data, achieving remarkable results in various natural language processing tasks.

### 4.2 Datasets

We selected a total of four representative datasets and conducted five experiments. Table 1 is a brief summary of these databases.

**Table 1:** Dataset basic information.

**Dataset**	**Number of class**	**Total number of data**
AGNews	4	30,000
Yahoo! Answers	10	140,000
R2	2	5,859
R5	5	6,803
CCTC dataset	22	45,452

AGNews (Zhang and LeCun, 2015): AGNews is a news classification corpus. It consists of four categories: World, Sports, Business, and Technology. Yahoo! Answers (Zhang and LeCun, 2015): Each text in this corpus includes a question and its corresponding answer. There are ten categories: Society and Culture, Science and Mathematics, Health, Education and Reference, Sports, Business and Finance, Entertainment and Music, Family and Relationships, Computers and Internet, Politics and Government.

Reuters-21578 Corpus: Initially collected and labeled by the Carnegie Group and Reuters, this corpus contains 21,578 documents categorized into 135 classes. Unlike other corpora, this dataset is highly imbalanced, with some classes having thousands of examples while others only have a few. Based on previous research (Cai et al., 2010), we selected two clustering corpora, R2 and R5, which include the two and five largest classes, respectively. The classes in corpus R2 are “earn” and “acq.” The classes in corpus R5 are “earn,” “acq,” “cude,” “trade,” and “money fx.” In the upcoming experiments, we utilize these imbalanced corpora to evaluate our model.

Government Complaint Telephone Content Dataset: We compiled a dataset consisting of 45,452 government complaint telephone content entries across 22 categories, including epidemic prevention and control, civil affairs, enterprise services, technology and information industry, education and sports, community cadres' conversations, rural agriculture, organizational personnel, labor and social security, land and resources management, party and government affairs, business and trade tourism, environmental protection inspection, economic management, environmental protection, discipline inspection and supervision, transportation, health and family planning, political and legal, urban and rural construction, and others. The numbers of instances vary, with the largest category being business and trade tourism with 22,518 entries, and the smallest being party and government affairs with 41 entries. The dataset content is similar to this: “The caller reported the loss of a mobile phone and identity card in Hall 3 of the XX Convention Center. They complained to the Convention Center Organizing Committee and the Committee's leader, Mr. Zhou, stating that there were no reminders for visitors to take care of their personal belongings. After citizens raised concerns, they also shifted responsibility. Please handle the matter.”

In the AGNews, Yahoo! Answers, and CCTC Dataset, we extracted 1000 samples for each class. This choice was made to achieve a small but balanced dataset, as it has been observed that a small yet balanced dataset can yield models similar to those trained on the original data (Wang et al., 2016). Sampling 1000 samples per class significantly reduces the computational load while maintaining a reasonable impact on model performance. In the R2 dataset, we randomly selected 2,000 instances from each class, resulting in a total of 4000 instances. For the R5 dataset, we added the remaining three classes to the R2 dataset, totaling 6,803 instances. For the English datasets, we performed simple preprocessing, which included merging all lines within a document into one, removing special symbols and stopwords, and performing lemmatization. As for the Chinese dataset, we conducted basic preprocessing such as tokenization and stopwords removal.

### 4.3 Evaluation metrics

We chose three evaluation metrics in total to assess the model performance using different criteria.

Clustering Accuracy (ACC) (Xu et al., 2003): The clustering accuracy is defined as follows:


(12) $$\begin{array}{l} ACC=\underset{m}{max}\frac{\sum_{i=1}^{n}1\{l_{i}=m(c_{i})\}}{n} \end{array}$$

where *l*_*i*_ is the true label of document i, *C*_*i*_ is the predicted label from the clustering algorithm, and m() is a one-to-one mapping between cluster labels and ground truth labels. The function 1{} outputs 1 when the equation inside the curly brackets is true and 0 otherwise.

Normalized Mutual Information (NMI) (Strehl and Ghosh, 2002): The normalized mutual information is given by:


(13) $$\begin{array}{l} \text{NMI}\left(L,C\right)=\frac{MI\left(L,C\right)}{\sqrt{H\left(L\right)H\left(C\right)}} \end{array}$$

where L represents the true labels and C represents the predicted labels from the clustering algorithm. MI(L, C) is the mutual information between L and C, measuring their relationship. NMI scales the MI(L, C) value between 0 and 1.

Adjusted Rand Index (ARI) (Yeung and Ruzzo, 2001): The Adjusted Rand Index is calculated as:


(14) $$\begin{array}{l} \text{ARI}=\frac{\sum_{i,j}\left(\begin{array}{c} n_{i,j}\\ 2 \end{array}\right)-\sum_{i}\left(\begin{array}{c} n_{i.}\\ 2 \end{array}\right)\sum_{j}\left(\begin{array}{c} n_{.j}\\ 2 \end{array}\right)/\left(\begin{array}{c} n\\ 2 \end{array}\right)}{\frac{1}{2}\left[\sum_{i}\left(\begin{array}{c} n_{i.}\\ 2 \end{array}\right)+\sum_{j}\left(\begin{array}{c} n_{.j}\\ 2 \end{array}\right)\right]-\left[\sum_{i}\left(\begin{array}{c} n_{i.}\\ 2 \end{array}\right)\sum_{j}\left(\begin{array}{c} n_{.j}\\ 2 \end{array}\right)\right]/\left(\begin{array}{c} n\\ 2 \end{array}\right)} \end{array}$$

where n is the total number of instances, *n*_*i, j*_ is the number of instances that appear in both the i-th predicted label and the j-th true label. ARI computes the similarity between ground truth labels and clustering algorithm predicted labels, and normalizes it between [0,1].

### 4.4 Results and discussion of the comparative analysis with other methods

The table provided above presents the results of all models on all five datasets. I, LN, N, MM stand for Identity Normalization, Layer Normalization, Standard Normalization, and Min-Max Normalization feature transformation strategies, respectively. For each dataset, three evaluation metrics are used to assess the clustering results. We ran each model 1,000 times and recorded the best results. As a result, we obtained 15 metrics for the four datasets.

As shown in Tables 2–5, our model outperforms classical bag-of-words models, word embedding models, and traditional clustering models based on pre-trained models, proving the effectiveness of the TCBPMA model. Specifically, the ELMO+Mean+LN+DEC model performs the best on all datasets. For instance, on the AGNews dataset, it achieves an accuracy of 84.4%, which is 3.7% higher than the best comparative method ELMO+K-means.

**Table 2:** Cluster evaluation on AGNews.

**Methods**	**ACC**	**NMI**	**ARI**
**AGNews**			
TFIDF+k-means	0.568	0.330	0.300
Word2Vec+k-means	0.567	0.485	0.494
ELMO+k-means (Guan et al., 2020)	0.807	0.591	0.582
DEC	0.470	0.124	0.115
TFIDF+DEC (Subakti et al., 2022)	0.721	0.386	0.414
Word2Vec+DEC	0.784	0.499	0.532
BERT+DEC (Subakti et al., 2022)	0.803	0.538	0.570
**TCBPMA**	**0.844**	**0.586**	**0.644**

The bold value indicates that the Acc value is the highest.

**Table 3:** Cluster evaluation on Yahoo! Answers.

**Methods**	**ACC**	**NMI**	**ARI**
**Yagoo! Answers**			
TFIDF+k-means	0.319	0.187	0.165
Word2Vec+k-means	0.227	0.344	0.263
ELMO+k-means	0.466	0.328	0.314
DEC	0.142	0.120	0.110
TFIDF+DEC	0.402	0.217	0.162
Word2Vec+DEC	0.439	0.326	0.192
BERT+DEC	0.475	0.290	0.233
**TCBPMA**	**0.524**	**0.370**	**0.275**

The bold value indicates that the Acc value is the highest.

**Table 4:** Cluster evaluation on R2.

**Methods**	**ACC**	**NMI**	**ARI**
**R2**			
TFIDF+k-means	0.739	0.348	0.348
Word2Vec+k-means	0.871	0.554	0.551
ELMO+k-means	0.843	0.498	0.484
DEC	0.760	0.374	0.369
TFIDF+DEC	0.859	0.506	0.515
Word2Vec+DEC	0.894	0.608	0.653
BERT+DEC	0.849	0.504	0.489
**TCBPMA**	**0.916**	**0.634**	**0.692**

The bold value indicates that the Acc value is the highest.

**Table 5:** Cluster evaluation on R5.

**Methods**	**ACC**	**NMI**	**ARI**
**R5**			
TFIDF+k-means	0.522	0.410	0.410
Word2Vec+k-means	0.129	0.583	0.623
ELMO+k-means	0.557	0.459	0.458
DEC	0.379	0.222	0.185
TFIDF+DEC	0.587	0.425	0.468
Word2Vec+DEC	0.713	0.542	0.535
BERT+DEC	0.723	0.549	0.529
**TCBPMA**	**0.760**	**0.554**	**0.614**

The bold value indicates that the Acc value is the highest.

Among all comparative models, BERT+DEC is one of the strongest, but it still lags behind our deep clustering model TCBPMA, especially on the R2 dataset. This is because although the BERT model excels in handling long texts and cross-text tasks, it may suffer from information scarcity when dealing with shorter texts. One possible reason is that the word embeddings of the ELMO model are context-dependent, while BERT's word embeddings are generated based on the entire sentence. For shorter texts, where context is limited, using the ELMO model can better capture the semantic information of individual words. The ELMO model can generate multi-level word vector representations, including lexical, sentence-level, and contextual levels. In short text clustering, using different levels of word vector representations helps capture diverse semantic information. BERT's training requires sentence pairs as input, which can be challenging to find for short texts. Therefore, in the clustering of shorter texts, ELMO's performance is better than BERT's, but for longer texts, BERT's performance is still superior to ELMO's, as shown in Table 6. Additionally, BERT demands extensive pre-training and consumes more computational resources and time. These factors contribute to our model's superior performance in short text clustering.

**Table 6:** Cluster evaluation on CCTC dataset.

**Methods**	**ACC**	**NMI**	**ARI**
**CCTC dataset**			
TFIDF+k-means	0.127	0.432	0.230
Word2Vec+k-means	0.242	0.283	0.181
ELMO+k-means	0.196	0.365	0.258
DEC	0.275	0.167	0.071
TFIDF+DEC	0.501	0.389	0.271
Word2Vec+DEC	0.465	0.353	0.244
**BERT+DEC**	**0.526**	**0.486**	**0.439**
TCBPMA	0.518	0.426	0.374

The bold value indicates that the Acc value is the highest.

Regarding the traditional clustering algorithm K-means, whether combined with feature-based text representations or pre-trained deep learning models, its performance is not as good as the deep clustering algorithm DEC. Especially on the R5 dataset, the accuracy difference is around 20%. While K-means is versatile and computationally efficient, it is highly sensitive to noise and outliers and does not perform well on high-dimensional data. Furthermore, short texts are inherently sparse due to their limited length, leading to noise and spelling errors, such as “4u” instead of “for you,” or “thx” instead of “thank you.” Hence, traditional clustering algorithms are not directly applicable to short text clustering. K-means's accuracy in short text clustering tends to be lower than in long text clustering. On the other hand, DEC is an end-to-end unsupervised deep clustering model that learns low-dimensional feature representations using autoencoders and performs clustering in a fine-tuning stage on those representations. This approach effectively addresses the challenges of high dimensionality and sparsity.

### 4.5 Results and discussion of ablation experiments

In this experiment, we conducted a comparison using four different feature normalization methods as ablation experiments.

In the neural language model, as shown in the previous section, two methods are used to fuse all features into fixed-sized features. From the Tables 7–11, it can be observed that average pooling yields better experimental results than max pooling. Compared to max pooling, average pooling can better capture the overall information of the text, rather than just focusing on local information. For a sentence, average pooling can better reflect the overall semantic meaning of the sentence, while max pooling only considers the most important words, potentially overlooking other important information. Additionally, average pooling is relatively more stable compared to max pooling, which can mitigate biases in word selection by the model.

**Table 7:** Evaluation of ablation experiments on AGNews.

**Methods**	**ACC**	**NMI**	**ARI**
**AGNews**			
ELMO+MAX+I+DEC	0.527	0.220	0.185
ELMO+MAX+N+DEC	0.264	0.005	0.004
ELMO+MAX+MM+DEC	0.250	0.000	0.000
ELMO+MAX+LN+DEC	0.823	0.550	0.590
ELMO+Mean+I+DEC	0.388	0.077	0.068
ELMO+Mean+N+DEC	0.250	0.000	0.000
ELMO+Mean+MM+DEC	0.395	0.097	0.072
**ELMO+Mean+LN+DEC**	**0.844**	**0.586**	**0.644**

The bold value indicates that the Acc value is the highest.

**Table 8:** Evaluation of ablation experiments on Yahoo! Answers.

**Methods**	**ACC**	**NMI**	**ARI**
**Yahoo! Answers**			
ELMO+MAX+I+DEC	0.340	0.201	0.134
ELMO+MAX+N+DEC	0.100	0.000	0.000
ELMO+MAX+MM+DEC	0.135	0.018	0.006
ELMO+MAX+LN+DEC	0.487	0.339	0.250
ELMO+Mean+I+DEC	0.132	0.014	0.007
ELMO+Mean+N+DEC	0.100	0.000	0.000
ELMO+Mean+MM+DEC	0.138	0.016	0.008
**ELMO+Mean+LN+DEC**	**0.524**	**0.370**	**0.275**

The bold value indicates that the Acc value is the highest.

**Table 9:** Evaluation of ablation experiments on R2.

**Methods**	**ACC**	**NMI**	**ARI**
**R2**			
ELMO+MAX+I+DEC	0.869	0.526	0.544
ELMO+MAX+N+DEC	0.500	0.000	0.000
ELMO+MAX+MM+DEC	0.743	0.185	0.235
ELMO+MAX+LN+DEC	0.894	0.602	0.621
ELMO+Mean+I+DEC	0.518	0.002	0.001
ELMO+Mean+N+DEC	0.500	0.000	0.000
ELMO+Mean+MM+DEC	0.517	0.002	0.001
**ELMO+Mean+LN+DEC**	**0.916**	**0.634**	**0.692**

The bold value indicates that the Acc value is the highest.

**Table 10:** Evaluation of ablation experiments on R5.

**Methods**	**ACC**	**NMI**	**ARI**
**R5**			
ELMO+MAX+I+DEC	0.530	0.423	0.363
ELMO+MAX+N+DEC	0.200	0.000	0.000
ELMO+MAX+MM+DEC	0.405	0.170	0.125
ELMO+MAX+LN+DEC	0.549	0.520	0.405
ELMO+Mean+I+DEC	0.405	0.018	0.030
ELMO+Mean+N+DEC	0.200	0.000	0.000
ELMO+Mean+MM+DEC	0.440	0.017	0.029
**ELMO+Mean+LN+DEC**	**0.760**	**0.554**	**0.614**

The bold value indicates that the Acc value is the highest.

**Table 11:** Evaluation of ablation experiments on CCTC dataset.

**Methods**	**ACC**	**NMI**	**ARI**
**CCTC dataset**			
ELMO+MAX+I+DEC	0.411	0.304	0.183
ELMO+MAX+N+DEC	0.125	0.000	0.000
ELMO+MAX+MM+DEC	0.352	0.228	0.135
ELMO+MAX+LN+DEC	0.417	0.290	0.180
ELMO+Mean+I+DEC	0.453	0.359	0.244
ELMO+Mean+N+DEC	0.125	0.000	0.000
ELMO+Mean+MM+DEC	0.476	0.348	0.220
**ELMO+Mean+LN+DEC**	**0.518**	**0.426**	**0.324**

The bold value indicates that the Acc value is the highest.

In our TCBPMA model, feature transformation is performed before inputting the feature vectors into the clustering algorithm. Layer normalization is the most effective strategy when configuring feature fusion based on average pooling. Compared to the configuration of ELMO+Mean+I+DEC, the configuration of ELMO+Mean+LN+DEC shows significant performance improvement. ELMO itself is a model with layer normalization, which uses residual connections and layer normalization to address the vanishing and exploding gradient problems in deep neural networks. Therefore, when using ELMO for text representation, layer normalization is more adaptive, allowing better retention of ELMO's features. On the other hand, the poor performance of min-max normalization might be due to the presence of outliers or anomalies in the data, which significantly affect the calculation of the minimum and maximum values, causing issues with the normalization of the entire dataset. Additionally, the simplicity of min-max normalization's calculation, without considering correlations across different feature dimensions, might also contribute to its performance decline. In contrast, layer normalization can better consider correlations across different feature dimensions, whereas identity normalization might not adapt well to various data distribution scenarios. In some cases, the value of NMI is zero. This could be because the feature vectors obtained by combining feature extraction with max pooling and feature normalization in the feature extraction module may not effectively capture useful information in the data, especially information related to clustering. In the clustering module, improper selection of hyperparameters for the DEC algorithm based on autoencoders may also lead to poor clustering results. Here, we used uniform hyperparameters, which could potentially result in an NMI of zero.

### 4.6 Results visualization

The experimental results from the previous section indicate that our clustering model outperforms traditional text clustering models based on features and generative models. This improvement can be attributed to the fact that the distributed text representations constructed by deep models position similar texts closer together, with the Euclidean distances between text features representing semantic relationships. To better visualize the experimental results, we employ the t-SNE visualization method (Van der Maaten and Hinton, 2008). t-SNE is based on probabilistic distributions of similarity to map data from high-dimensional space to a lower-dimensional space, preserving the relative distances between similar data points in the lower-dimensional space and retaining the local structure of the original data as much as possible.

**Figure 5:**
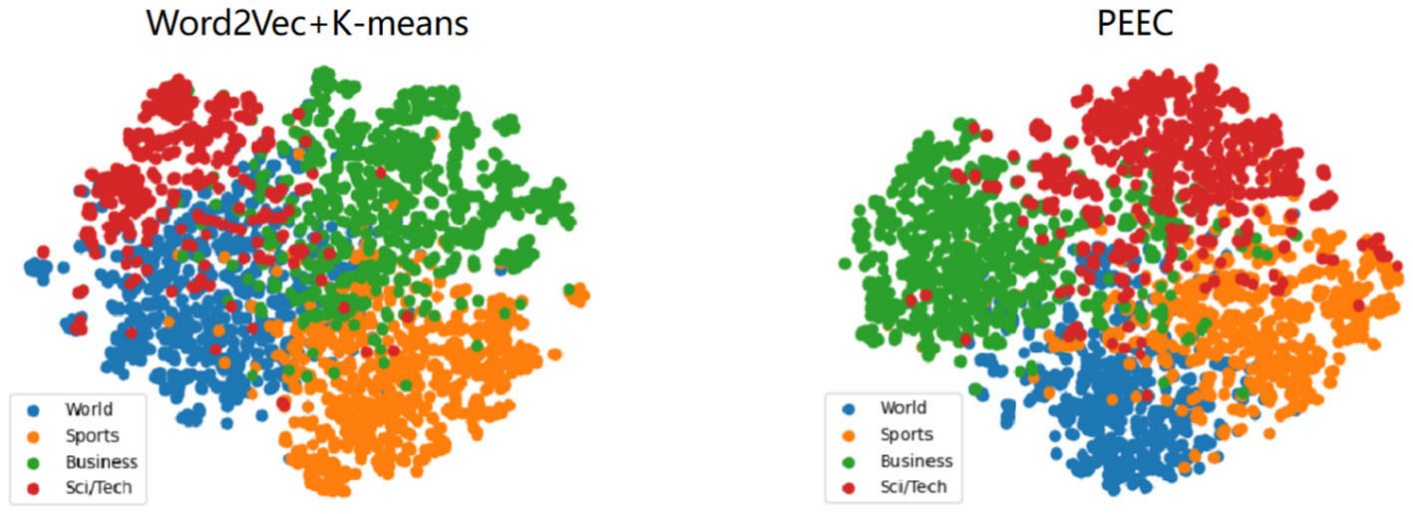
AGNews visualization.

**Figure 6:**
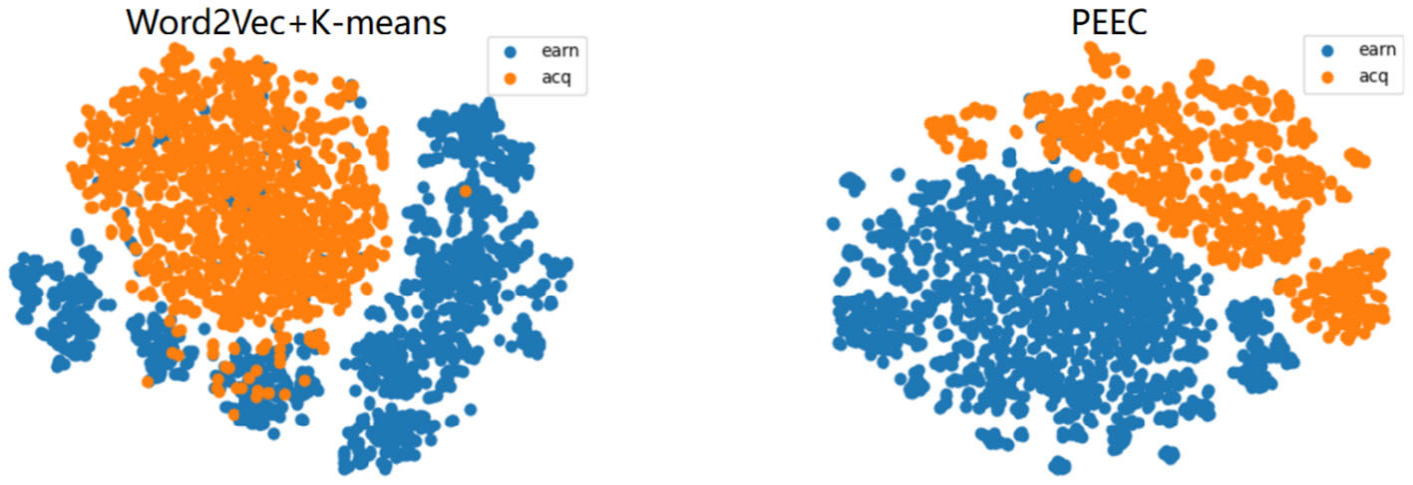
R2 visualization.

For visualization in a two-dimensional space, we set the output dimensions of t-SNE to 2. We select the AGnews and R2 datasets for feature visualization.The results are as shown in Figures 5, 6. After referring to the original t-SNE paper, we set the random seed to 30, the learning rate to 200, and the number of iterations to 1,000. We experimentally test a range of perplexity values from 5 to 50 and ultimately choose 30 based on the visualization results. The left figure shows the visualization result of word2vec+K-means, and the right figure shows the visualization result of our TCBPMA model. It's evident that our model performs better in text clustering. In Figure 1, blue, orange, green, and red represent World, Sports, Business, and Sci/Tech categories respectively. In Figure 2, blue represents the “earn” category, and orange represents the “acq” category.

### 4.7 Nemenyi test graph analysis

**Figure 7:**
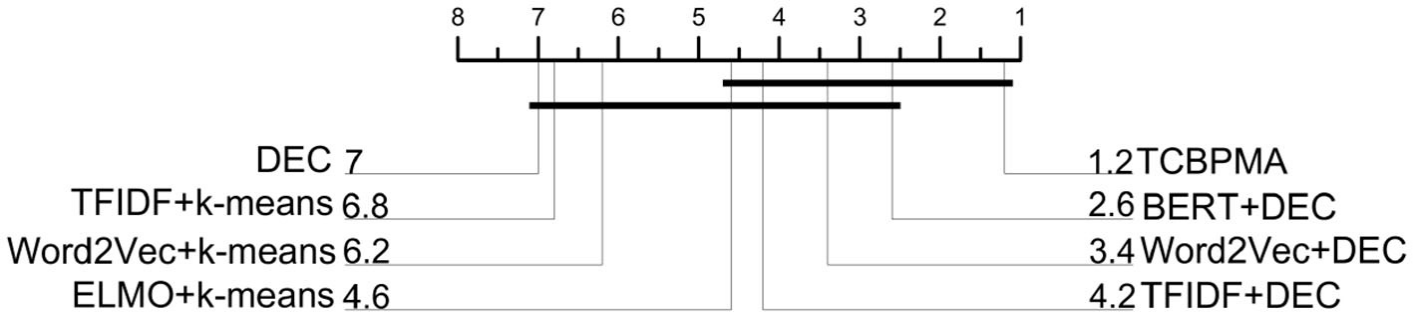
Nemenyi test graph.

We extracted precision values of different methods across five datasets and employed MATLAB to generate the Nemenyi test plot. The results are as shown in Figure 7. Through the analysis of this plot, our framework's advantages became more evident, with our approach ranking first, followed by BERT+DEC, and the original DEC performing the least effectively.

## 5 Conclusion

This article introduces a deep learning-based text clustering framework that combines pre-trained models with deep clustering algorithms, effectively enhancing the accuracy and efficiency of text clustering. Experimental results demonstrate that this model performs exceptionally well across various datasets and exhibits greater robustness compared to traditional text clustering algorithms. Furthermore, this model is flexible and scalable and can be applied to medical data clustering. The proposed framework has some limitations. For instance, the framework relies on context and may not be sufficiently adaptable to dynamic or continuously changing data streams. When handling real-time or dynamic data, additional mechanisms may be necessary to maintain the stability of the model. Additionally, our model framework involves several adjustable hyperparameters, such as the number of clusters and learning rate. The selection of hyperparameters can significantly impact the performance of the framework, but identifying suitable hyperparameters typically requires extensive experimentation and tuning.

In the medical field, our clustering framework can be utilized for disease diagnosis. By analyzing patients' medical records, our clustering framework classifies patients, assisting doctors in more accurate disease diagnoses. For instance, patients can be categorized into different disease classes such as flu, pneumonia, gastritis, etc., based on their medical history, symptoms, and examination results. Additionally, it can contribute to drug development by analyzing drug components and mechanisms of action. This aids researchers in discovering new drug targets and combinations. Moreover, the framework can be employed to screen potential drug candidates, enhancing the efficiency of drug development.

In other industries, our clustering framework finds diverse applications. In the financial sector, it can analyze customer credit risks, assisting financial institutions in categorizing and managing clients. In marketing, through analyzing consumer purchasing behavior and preferences, our clustering framework helps businesses identify different consumer segments, enabling the formulation of more effective marketing strategies.

## Data availability statement

The raw data supporting the conclusions of this article will be made available by the authors, without undue reservation.

## Author contributions

QX: Conceptualization, Data curation, Funding acquisition, Investigation, Methodology, Software, Supervision, Writing – original draft, Writing – review & editing. HG: Conceptualization, Data curation, Formal analysis, Methodology, Project administration, Supervision, Validation, Writing – original draft, Writing – review & editing. SJ: Funding acquisition, Project administration, Resources, Supervision, Visualization, Writing – review & editing.
